# Vaginal dysbiosis and the potential of vaginal microbiome-directed therapeutics

**DOI:** 10.3389/frmbi.2024.1363089

**Published:** 2024-10-11

**Authors:** Valerie Diane Valeriano, Emilia Lahtinen, In-Chan Hwang, Yichan Zhang, Juan Du, Ina Schuppe-Koistinen

**Affiliations:** ^1^ Centre for Translational Microbiome Research (CTMR), Department of Microbiology, Cell and Tumor Biology, Karolinska Institutet, Stockholm, Sweden; ^2^ Gedea Biotech, Medicon Village, Lund, Sweden

**Keywords:** dysbiosis, vaginal microbiome, vaginal health, live biopharmaceutical products, lactobacilli

## Abstract

A healthy vaginal microbiome (VMB) is dominated by *Lactobacillus* spp. and provides the first line of defense against invading pathogens. Vaginal dysbiosis, characterized by the loss of *Lactobacillus* dominance and increase of microbial diversity, has been linked to an increased risk of adverse genital tract diseases, including bacterial vaginosis, aerobic vaginitis, vulvovaginal candidiasis, sexually transmitted infections, and pregnancy complications such as preterm birth. Currently, antibiotics and antifungals are recommended first-line treatments with high cure rates, but they also can lead to high recurrence and resistance development. As an alternative, lactobacilli have been utilized to restore the vaginal microbiota. In this review article, we discuss vaginal dysbiosis in various gynecological infections and potential interventions based on Live Biotherapeutic Products (LBPs) with a focus on those that use intravaginal treatment modalities to modulate the VMB. Based on these, we provide insights on key factors to consider in designing phenotypic and genotypic screens for selecting bacterial strains for use as vaginally administered microbiome-directed therapeutics. Lastly, to highlight current progress within this field, we provide an overview of LBPs currently being developed with published clinical trial completion for recurrent BV, VVC, and UTI. We also discuss regulatory challenges in the drug development process to harmonize future research efforts in VMB therapy.

## Introduction

The vaginal microbiome (VMB) is composed of all the microorganisms inhabiting the vagina. The composition of the vaginal microbiome is dynamic, changing throughout a woman’s lifespan, with notable shifts occurring during hormonal changes, such as menstruation, pregnancy, and menopause. Ravel et al. (2011) performed one of the earliest in-depth analyses of the VMB in reproductive-aged women of varying ethnic groups using next-generation sequencing. They proposed a classification based on Community State Types (CSTs), where the CSTs and their subtypes describe the temporal changes in the composition of the VMB (Ravel et al., 2011; Holm et al., 2023). CSTs I, II, III, and V are characterized by the dominance of different *Lactobacillus* species, such as *L. crispatus*, *L. gasseri*, *L. iners*, and *L. jensenii*, respectively. Healthy reproductive-aged females are generally CST-I (*L.crispatus*-dominated) and CST III (*L. iners*-dominated). In contrast, CST IV and its subtypes characterize VMBs dominated by facultative anaerobes, such as *Gardnerella vaginalis* and *Fannyhessea vaginae* (formerly *Atopobium vaginae)*, resulting in a high-diversity vaginal microbiome with a reduced abundance of lactobacilli. Current prospective studies on reproductive outcomes of young, healthy women confirm the CSTs classification (Haahr et al., 2016; Kroon et al., 2018). However, due to the dynamic nature of the human VMB and the complexity of the various state types, not all women neatly fit into each CST. A modular approach was proposed in a Belgian cohort describing the co-occurrence of bacterial taxa based on network correlation analysis (Lebeer et al., 2023), where common species associated with bacterial vaginosis (BV) and a healthy VMB can be present in asymptomatic women. In addition, some women, especially those of Black or Hispanic background, may have a natural VMB dominated by non-*Lactobacillus* species. As more research is performed on defining a healthy VMB, more detailed sub-types based on the VAginaL community state typE Nearest CentroId clAssifier (VALENCIA) have been proposed. VALENCIA separates CST III and CST IV groups based on the relative abundance of different bacterial species (France et al., 2020). In addition, metagenomic community state types (mgCSTs) enable the separation of the same species based on the assembled metagenomes and functional diversity (Holm et al., 2023). Nevertheless, in all these findings, it is agreeable that a healthy VMB contributing to positive reproductive outcomes harbors a low-diversity microbial community dominated by different members of the *Lactobacillus* genus.

Furthermore, as our understanding of the VMB increases, it has been determined that vaginal dysbiosis that progresses from CST IV and its subtypes render women more susceptible to other infection-related issues, including BV (France et al., 2020), aerobic vaginitis (Donders et al., 2017), and sexually transmitted diseases (Edwards et al., 2019; Cheng et al., 2020; Wang et al., 2023). Vaginal dysbiosis also potentially increases the risk of other gynecological conditions associated with adverse pregnancy outcomes, such as miscarriage and preterm birth (Grewal et al., 2022; Gudnadottir et al., 2022).

A deep understanding of the composition and function of the VMB and its interaction with pathogens and the host immune system is necessary for the development of novel treatment options for vaginal dysbiosis. Recently, live biotherapeutic products (LBPs) have emerged as a promising and innovative class of therapeutic agents with broad applications, including addressing issues related to the VMB. The United States Food and Drug Administration (FDA) defines LBPs as biological products containing living organisms, such as bacteria, designed to prevent, treat, and cure human diseases or conditions, excluding vaccines. We aim to highlight key factors that can be important to delineate between probiotics with limited or no therapeutic claims as compared to these LBPs designed as drugs by revisiting the currently known consequences of vaginal dysbiosis that need attention and discuss the recent advancements in the application of LBPs for common gynecological conditions. Further, we outline the desirable characteristics of LBPs in treating vaginal dysbiosis and describe the challenges linked to LBP development for vaginal health.

## Vaginal dysbiosis and infectious causes of gynecological inflammation

**Figure 1:**
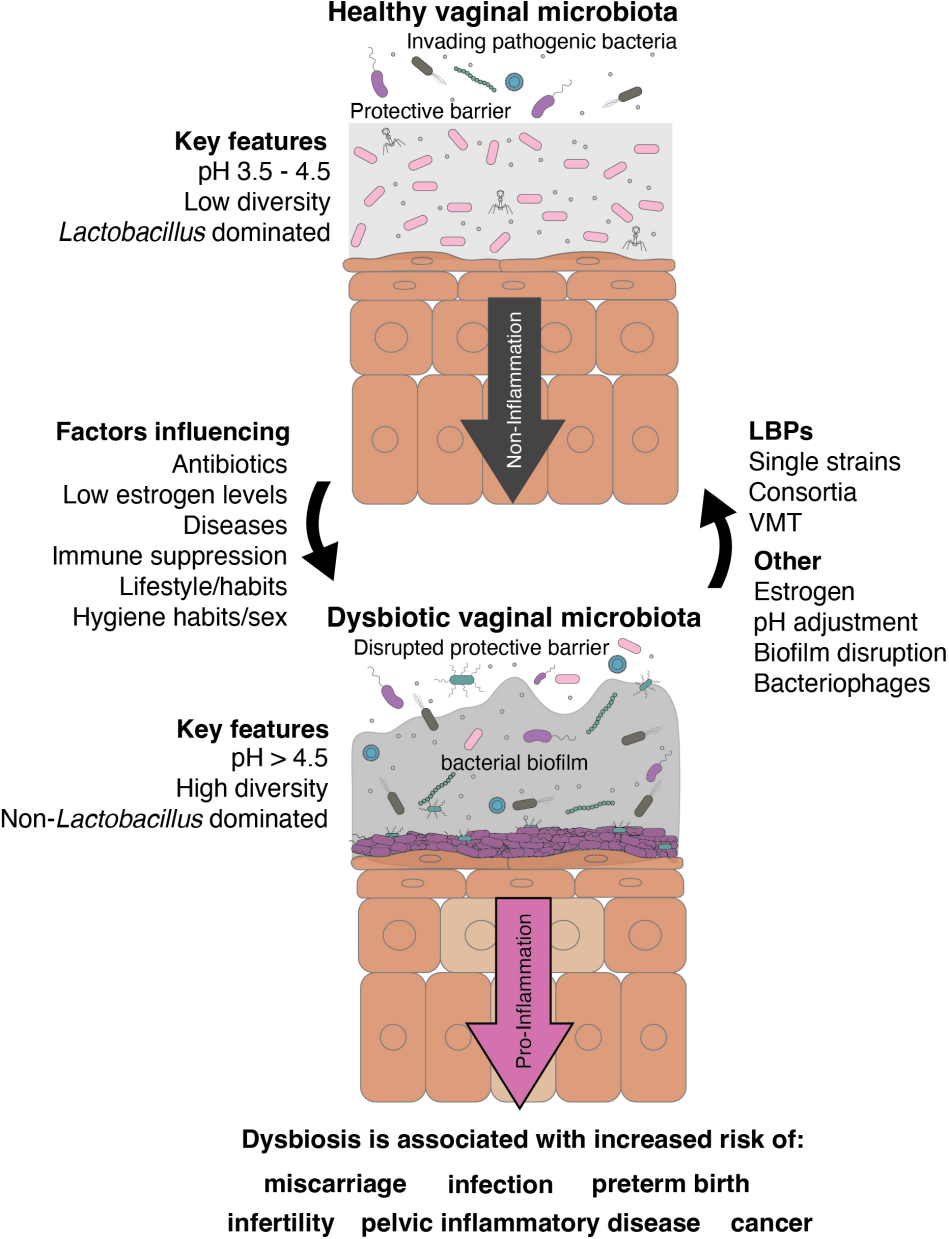
Graphical summary of the role of native lactobacilli in the vaginal tract and their potential role as LBPs in vaginal dysbiosis.

In a healthy vagina, the vaginal microenvironment harbors immune cells, such as natural killer cells, macrophages, and dendritic cells, abundance of which increases during inflammation (Monin et al., 2020; Anahtar et al., 2015). Lactobacilli are crucial for maintaining the vaginal mucosal layer’s integrity as a physical defense against pathogens. However, vaginal dysbiosis occurs depending on both biological and behavioral factors. Sexual activity may be a primary cause of some of these infections. However, even sexually inactive individuals can experience dysbiosis in their lifetime due to the use of antibiotics, immunosuppression, low estrogen levels, or unsuitable hygiene habits (**Figure 1**). Vaginal dysbiosis is generally characterized by a long-term high-diversity state, where non-lactobacilli members of the vaginal community flourish. In a dysbiotic vaginal microbiome, increased microbial diversity, along with a decrease in beneficial lactobacilli, is accompanied by immunomodulatory changes that affect the natural barrier and contribute to further alterations in the microbiome, vaginal homeostasis, and host immunity.

### Bacterial vaginosis

The most common dysbiotic state in the vagina occurs with BV, especially in reproductive-aged women worldwide. Its risk for susceptibility to other obstetric and gynecological diseases, such as preterm birth and sexually transmitted infections (STIs), renders a high global economic burden fueling the impetus for screening and therapeutic studies (Peebles et al., 2019). Nugent scoring based on microscopic evaluation of Gram-stained vaginal smear samples provided standardized measures for assessing vaginal health (Nugent et al., 1991; Coleman and Gaydos, 2018). Although the Nugent scoring is proposed as a gold standard and allows comparability between ethnic groups, it requires time and expertise, for which the Amsel criteria is more preferred especially in resource-limited settings (Bhujel et al., 2021). Clinically, the Amsel criteria are more commonly used, where 3 out of the 4 conditions are met for a diagnosis: 1) thin, homogenous discharge; 2) pH of vaginal fluid above 4.5; 3) clue cells on microscopy (more than 20% of epithelial cells); 4) positive KOH test (Amsel et al., 1983; Donders, 2010). However, given the lower specificity and sensitivity of the Amsel criteria (37 to 70%) as compared to Nugent scoring (94-97%) (CDC, 2021), variations in clinical diagnosis can affect the understanding of the contribution of ethnic differences to the prevalence of the condition and the etiology of the disease relevant to the microbiome.

First-line treatment for BV is mainly clindamycin or metronidazole, with cure rates decreasing from >90% (Joesoef et al., 1999) to 50-80% (Eschenbach, 2007) in recent reports of known BV cases, accompanied by high recurrence rates (Bradshaw et al., 2006; Coudray and Madhivanan, 2020; Verwijs et al., 2020). The variability in response to antibiotic treatment may be due to differences in how efficient the inconsistency the BV-associated biofilm is disrupted (Bradshaw and Sobel, 2016; Wu et al., 2022; Sousa et al., 2023). Further, within biofilms, BV-associated bacteria may possess inherent antibiotic resistance mechanisms, such as pumping, chemical neutralization, and matrix barriers (Hardy et al., 2017; Nourbakhsh et al., 2022). This poor clearance can possibly lead to a high recurrence rate, where more than half of the women experience BV symptoms again within one year of the antibiotic treatment and likely enrichment of antibiotic-resistance genes in the vaginal environment (Beigi et al., 2004a; Bradshaw et al., 2006).

During BV, the vagina fosters a pro-inflammatory environment, which can be partly attributed to the immunomodulatory effects of the anaerobic bacteria and their metabolites. Notably, short-chain fatty acids (SCFAs) are elevated during BV and their abundance is associated with increased inflammation (Delgado-Diaz et al., 2020; Schwecht et al., 2023). The presence of SCFAs such as acetate, propionate, butyrate, and succinate combined with biogenic amine production in the absence of lactobacilli increases vaginal pH >4.5 (O’Hanlon et al., 2011), making it favorable for growing anaerobic bacteria that produce virulence factors that can degrade mucin, compromise the vaginal epithelial barrier integrity, and stimulate pro-inflammatory responses (Aldunate et al., 2015). Further, the recognition of bacterial cells by the toll-like receptors (TLRs) on the vaginal epithelial cells launches an inflammatory response (Aldunate et al., 2015; Delgado-Diaz et al., 2020). Aside from the production of amines, recent research found that specific amino acid metabolites secreted by BV-associated bacteria, such as imidazole propionate, can interfere with vaginal epithelial cell function and further contribute to inflammation via the mTOR signaling pathway (Berard et al., 2023).

Within the context of the vaginal microbiome, the molecular and pathophysiological mechanisms of BV development remain largely unknown. What is known so far is that women suffering from BV are characterized by a dysbiotic vaginal microbiome dominated by a diverse group of anaerobic bacteria, such as *Gardnerella vaginalis*, *Fannyhessea vaginae*, *Prevotella bivia, and Sneathia amnii* (Fredricks et al., 2005; Muzny et al., 2019; Carter et al., 2023). The VMB is also depleted of *Lactobacillus* spp., except for *L. iners*, often found adapting and coexisting with BV-associated bacteria (Zheng et al., 2021). *G. vaginalis* and *Prevotella* have been touted as primary instigators (Machado et al., 2013; Muzny et al., 2018, 2019; van Teijlingen et al., 2024) of BV and biofilms are formed with other important anaerobic bacteria during pathogenesis (Bradshaw and Sobel, 2016; Hardy et al., 2017). Due to the nature of the polymicrobial infection, metabolic environmental shifts lead to continued host inflammatory responses and disrupted epithelial cell barrier, which become further aggravated, leading to secondary infections and adverse gynecological disease sequelae.

### Aerobic vaginitis

Aerobic vaginitis, due to its similarity to BV, can be misdiagnosed and incorrectly treated. It has been associated with complications such as vaginal inflammation, bacteriuria, lower urinary tract infections, and acute pyelonephritis (Kaambo et al., 2018). AV is characterized by an increase in enteric aerobic commensals not generally found in BV, such as group B *Streptococcus agalactiae* (GBS), *Staphylococcus aureus*, *Escherichia coli*, and enterococci, in the vaginal microbiome (Donders et al., 2017). Compared to the normal vaginal flora, these aerobic bacteria increase by 3- to 5-fold and are associated with vaginal mucosal inflammation due to the disruption of *Lactobacillus* dominance (Fan et al., 2013). The main symptoms are similar to those of BV but present as negative on the KOH test (Donders et al., 2015). Severe inflammation is apparent in vaginal smears, where an increase in intermediate and basal cells occurs. It is accompanied by increased turnover of the superficial epithelial cell layer and possibly epidermal desquamation, vaginal epithelial atrophy, small erosions, and ulceration (Petrova et al., 2015). Immune system dysregulation is the main factor contributing to AV pathogenesis (Oerlemans et al., 2020a). Similarly, reduced estrogen levels and localized immunity make AV prevalent in postmenopausal women (Stika, 2010). Pathogenic members of AV, such as GBS, are an essential focus, as they can occasionally cause morbidity in older adults, pregnant women, and patients with underlying medical conditions (Kwatra et al., 2014; Brigtsen et al., 2015). GBS colonization of pregnant women is considered a significant cause of severe neonatal infections, including neonatal sepsis, meningitis, and pneumonia (Krauss-Silva et al., 2014). In addition to GBS, *Enterococcus faecalis* has also been associated with preterm birth, low birth weight, and puerperal sepsis, resulting in significant maternal and neonatal morbidity and mortality (Cools et al., 2016; Kim et al., 2014).

As such, preferred treatments for AV are kanamycin and quinolones, which have intrinsic activity against disease-causing pathogenic bacteria while potentially minimizing interference with the vaginal microbiota (Tempera and Furneri, 2010). So far, the use of meclocycline and kanamycin for the treatment of AV allows a focus on Gram-negative bacilli rather than Gram-positive cocci, as these drugs are not absorbed and may leave extra vaginal lactobacilli even in patients with severe AV (Tempera and Furneri, 2010). Likewise, clindamycin for treating BV is also used for AV treatment due to its broad spectrum of activity against several aerobic Gram-positive coccal species; however, infection control with clindamycin is short-lived and may not cover all species associated with AV (Sousa et al., 2023). On the other hand, non-absorbable, broad-spectrum antibiotics such as carbapenems and clavulanic acid-beta-lactam combination (amoxiclav) are very effective for rapid, short-term improvement of severe symptoms from aerobic infections, especially deep cutaneous vulvovaginitis caused by GBS or methicillin-resistant *S. aureus* (MRSA) (Donders et al., 2015). Some beta-lactam antibiotics, however, are associated with less efficacy in eradicating *E. coli* vaginal colonization, leading to a high frequency of recurrent urinary tract infections (Stapleton, 2016). Nevertheless, due to significant inflammation in AV patients, including leukocyte and parabasal cell infiltration, frequent and prolonged use of antibiotics may result in adverse side effects. It may need to be accompanied by local administration of estrogens or a combination of probiotics and a very low dose of local estriol for postmenopausal or immunocompromised patients (Donders et al., 2015).

### Vulvovaginal candidiasis

Like BV and AV, VVC is another significant public health concern. In the US alone, more than 70% of women experience fungal infection at least once in their life, with inflammation of the vagina and vulva as the primary concern (Nyirjesy et al., 2022). They are commonly colonized in the vagina by *Candida albicans* and non-albicans species such as *C. glabrata*, *C. tropicalis*, *C. krusei*, and *C. parapsilosis*. Other risk factors for VVC include antibiotic usage, lifestyle choices such as sexual activity, serum glucose levels in diabetic and pre-diabetic females, estrogen balance, immunoregulatory deficiencies, allergies, and gene polymorphisms (Satora et al., 2023). Thus, VVC classifications help to form treatment modalities based on uncomplicated and complicated VVC. Those with uncomplicated VVC are infrequent, with mild to moderate symptoms. *Candida albicans* is usually the main culprit in uncomplicated VVC. On the other hand, non-albicans candidiasis mainly occurs in those who are immunocompromised, leading to more severe and recurrent VVC (Nyirjesy et al., 2022).

Immunity against candidiasis is complex and involves multiple processes, including pathogen recognition through receptors such as Toll-like receptors (TLR) and C-type lectin receptors (CLR), active innate and adaptive immune cells such as macrophages, dendritic cells (DC), and T cells, as well as the production of cytokines and chemokines such as IL-4, IL-6, IL-10, IL-12, IFN-γ, and TNF-α (Netea et al., 2015; Pathakumari et al., 2020; Zhou et al., 2021a). A prospective study found higher serum levels of cytokines, including IL-4, IL-6, and IL-10, and lower levels of IFN-γ, TNF-α, and IL-17F in patients with recurrent VVC compared to those with VVC. This indicates that Th1/Th2 immunity may play an essential role in VVC (Ge et al., 2022). The host immune response against candidiasis has been comprehensively summarized in other reviews and will not be discussed in detail in this review (Netea et al., 2015; Pathakumari et al., 2020; Zhou et al., 2021a).

However, *Candida* spp. has evolved mechanisms for immune evasion and dissemination by which it can hijack macrophages and instigate hyphal escape via pyroptosis (Uwamahoro et al., 2014) and macrophage extracellular trap formation (ETosis) (Netea et al., 2015; Olivier et al., 2022). To escape immune detection, *Candida* spp. executes morphological switching from non-infectious yeast to hyphae inside the macrophages. It instigates hyphal escape by producing pore-forming proteins such as candidalysin (Olivier et al., 2022), leading to inflammation and macrophage rupture, consequently releasing the pathogen. Further, *Candida* spp. can neutralize acidic growth environments by releasing ammonia derived from the catabolism of amino acids that may promote evasion and survival, although regulated by mitochondrial respiration (Westman et al., 2018; Silao et al., 2024). Other non-canonical strategies promoting colonization and evasion include β-glucan masking, which is triggered by stressors, nutrients, antifungal drugs, and other factors such as nitrogen availability and quorum sensing molecules (Pradhan et al., 2019). Such adaptations may contribute to the possibility of recurrence aside from other factors that can promote or induce VVC.

Even in healthy females, *Candida albicans* can co-exist as commensal members of the *Lactobacillus*-dominated VMB. Vaginal lactobacilli are known to produce anti-*Candida* factors, specifically with growth inhibition and targeting of hyphal components. Nevertheless, it has been reported that candidiasis occurs more frequently with an *L. iners*-dominated microbiome (CST III) as compared to an *L. crispatus*-dominated flora (CST II) (Tortelli et al., 2020). *L. crispatus* can inhibit the growth of *C. albicans* through the production of lactic acid and other anti-*Candida* molecules (Jang et al., 2019) and has the potential to reduce hyphal morphogenesis (Wang et al., 2017). Lactobacilli can further inhibit *C. albicans* virulence by preventing fungal adhesion to vaginal epithelial cells and hindering biofilm formation (Takano et al., 2023). *Candida* has been found to co-occur with high abundance of lactobacilli and BV-associated bacteria (Liu et al., 2013). *Candida* colonization is more commonly asymptomatic and may not always progress to VVC (Tortelli et al., 2020). However, some studies have implicated lactobacilli-rich VMBs associated with symptomatic VVC (Beigi et al., 2004b; McClelland et al., 2009). Nevertheless, with focus on the species level of lactobacilli, it has been more linked to the presence of an *L. iners*-rich VMB than an *L. crispatus*-rich VMB (Tortelli et al., 2020). In this context, the exact mechanism leading to the switch between commensal and pathogenic forms of *C. albicans* is not fully understood, especially in the context of *Lactobacillus-Candida* interactions in the vaginal environment (Takano et al., 2023; Silao et al., 2024).

Azole antifungal agents are used as first-line treatment, leading to adverse consequences such as the rise in multidrug-resistant *Candida* spp. Thus, despite standard treatment, the high prevalence of VVC and yeast colonization still puts this high on the World Health Organization’s (WHO) “critical priority” group. A review of evidence conducted by Nyirjesy et al. (2022) identifies the need for novel treatment approaches for recurrent VVC where no alternative treatments, even with oral probiotics, have solid evidence for support yet. Nevertheless, oteseconazole, another antifungal drug that finished a phase 3 study, presents a new option for recurrent VVC with fewer adverse effects that was approved in the USA for women who are not of reproductive potential (Martens et al., 2022; Siddiqui et al., 2023).

## Vaginal dysbiosis and the susceptibility to sexually transmitted diseases

A healthy, vaginal epithelium is usually highly protective against STIs caused by viruses such as HPV and HIV, parasites such as *Trichomonas vaginalis*, and bacteria such as *Neisseria gonorrhoeae*, *Mycoplasma genitalium*, and *Chlamydia trachomatis.* In the study of HIV infection, the vagina has been thought to be a markedly more effective barrier than the rectum. Many layers of stratum corneum cells are shed each day, thereby reducing the ability of pathogens to reach target cells deeper in the epithelium. Further, along with the inhibitory properties of the resident vaginal lactobacilli, epithelial and immune cells constitutively produce low levels of antimicrobial peptides (e.g., SLPI) and cytokines such as the anti-inflammatory IL-1RA that contribute to homeostasis (Cone, 2014; Muzny et al., 2020).

However, BV and microbial diversity can modify the risk of STIs via their interaction with mucosal immunity within the female genital tract and modification of its protective epithelial barrier (Velloza and Heffron, 2017). Dysbiosis-associated bacteria like *Gardnerella* spp. and *Prevotella* spp. can break down the mucosal barrier through sialidase production and host cell lysis, making women more susceptible to gynecological infections (France et al., 2022). *In vivo*, this microbial shift triggers a local immune response and inflammation, reflected in increased pro-inflammatory cytokine production of interleukin (IL)-1α, IL-1β, IL-6 and IL-8 in the vagina with BV-associated bacteria in CST IV such as *P. amnii*, *Sneathia* sp. and *Mobiluncus mulieris* as compared to *Lactobacillus*-dominated communities (Marconi et al., 2014; Anahtar et al., 2015). *In vitro*, cervical epithelial cells produce higher concentrations of IL-6 and IL-8 when co-cultured with *G. vaginalis*, *P. bivia*, and *P. amnii* compared to *L. crispatus*, whereas *F. vaginae* also elevates IL-6, IL-8, and TNF-α cytokines (Doerflinger et al., 2014).

### Human immunodeficiency virus

BV contributes to the proinflammatory cytokines in the vaginal tract of HIV-infected women (Mitchell et al., 2008). However, with these inflammatory states and disrupted barriers, infectious particles such as HIV can traverse through the genital epithelium via tears in the squamous epithelium or transcytosis across the single cell layer of the endocervix, ultimately infecting underlying CD4+ target cells in the submucosa (Vyshenska et al., 2017). The net outcome of these interactions favors HIV infection and replication by attracting target cells, which will subsequently become infected and further propagate the infection.

Recent publications have demonstrated that women with non-*Lactobacillus*-dominant VMB were at a greater risk of HIV infection, where increased mucosal inflammation enhanced the rate of sexual transmission of HIV in the female genital tract (Vitali et al., 2017; Wessels et al., 2018; Wang et al., 2023). On the other hand, *L. crispatus* is associated with reduced inflammation due to an immunoregulatory environment, exclusion of BV-associated bacteria, and reduced HIV risk. However, *L. iners* has been associated with fewer immune benefits and lower HIV protection or exclusion of BV-associated bacteria, possibly providing a more intermediate transitional stage to dysbiosis (Armstrong and Kaul, 2021). It is possible that women can develop a persistent long-term state where BV is frequent and that this state is associated with more frequent HIV infection. BV is more prevalent and persistent among HIV-infected women, particularly among those who are immunocompromised (Jamieson et al., 2001; Schellenberg et al., 2012).

### Human papillomavirus

A dysbiotic VMB poses similar risks for the increase in HPV infection and persistence (Mitra et al., 2016; Vyshenska et al., 2017; Muzny et al., 2020). CST-III (*L. iners*-dominated) and CST-IV (dominated by facultative anaerobes) are suggested to be risk factors for persistent HPV infection (Mei et al., 2022) due to low protective immunity with the loss of healthy vaginal lactobacilli. In addition, HPV proteins E6 and E7 enhance IL-10 expression and macrophage type 2 production (Kremleva and Sgibnev, 2016). Cytokines such as IL-6, IL-8 and TGFβ-1 could also potentially influence the growth of different vaginal microbes (Kremleva and Sgibnev, 2016).

During this time, strict anaerobic bacteria start to colonize the vagina and destroy the protective barrier of the cervical epithelium. This event promotes HPV entry of host cells, paving the way for subsequent integration of the HPV virus into the host DNA and accompanying cell division for virus replication. Nevertheless, a *Lactobacillus*-dominated VMB, especially with *L. crispatus* (Cheng et al., 2020), protects against HPV and prevents HPV persistence and subsequent disease development (Zhang et al., 2018; Muzny et al., 2020). In another study, an *L. gasseri*-dominated microflora (CST II) is also associated with faster HPV clearance (Mei et al., 2022). Without these protective mechanisms in place, the immune system cannot cope with the strong immunological pressure brought about by dysbiosis, allowing tumor evasion strategies that lead to cervical intraepithelial neoplasia or cervical cancer development in unresolved HPV infections (Piersma, 2011; Zhou et al., 2021b).

### *Trichomonas vaginalis* infection

*Trichomonas vaginalis* infection is associated with an increased risk of HIV infection and cervical and prostate cancer (Dessì et al., 2019; Margarita et al., 2020). The vaginal microbiota also plays a role in mediating susceptibility to *T. vaginalis* parasite infection, increasing the risk of trichomoniasis among women with BV or a Nugent score > 3 (Hinderfeld et al., 2019; Balkus et al., 2014; Margarita et al., 2020). Here, the loss of lactobacilli leads to an increased pH, creating an environment more favorable for *T. vaginalis* growth and pathogenicity (Margarita et al., 2020). Studies have also demonstrated an association between prevalent trichomoniasis and detection of *Prevotella* and *Sneathia* (non-*amnii*) species. These bacterial species may mediate susceptibility by producing nicotinamide metabolites that promote *T. vaginalis* infection (Jarrett et al., 2019). In contrast, suppression of *L. iners* in the initial interaction with *T. vaginalis* occurs but shows the possibility of adapting and surviving after longer exposure to *T. vaginalis* (Chiu et al., 2021). According to this relationship, interventions that decrease BV incidence and promote eubiosis could potentially contribute to reductions in trichomoniasis incidence (Balkus et al., 2014).

## Vaginal dysbiosis and its effect on adverse pregnancy outcomes, infertility, and IVF failure

Dysbiosis-induced cervicovaginal inflammation has been strongly linked to adverse pregnancy outcomes such as preterm birth (Fettweis et al., 2019). The most common infectious diseases correlated with pregnancy complications are BV, VVC, and AV (Gao et al., 2021). The mechanism for which VVC during pregnancy is still speculative (Bagga and Arora, 2020), but pregnancy-related factors such as elevated levels of estrogen and progesterone, glycogen deposition, low vaginal pH, and decreased immunity that affect the vaginal milieu remain risk factors for VVC (Tellapragada et al., 2017; Gao et al., 2021). This is observed in a prevalence study of 1119 pregnant women in different regions of the world, of which 30% is colonized by vaginal *Candida* based on culture (Disha and Haque, 2022). Despite the lack of precise mechanisms, the weakened immunity state of the female cervicovaginal tract during pregnancy remains conducive to *Candida* infection that can instigate prostaglandin release, leading to uterine contractions and premature birth. However, several large cohort studies and systematic reviews did not show any association between VVC and preterm birth (Schuster et al., 2020; Blomberg et al., 2023; Gigi et al., 2023).

AV, on the other hand, is characterized primarily by a high abundance of GBS and *E. coli*, which in severe cases may be accompanied by desquamative inflammatory vaginitis, is linked to pregnancy complications such as ascending chorioamnionitis, early membrane rupture, and preterm delivery (Donders et al., 2002, 2011; Ma et al., 2022). A significant reduction in the incidence of preterm birth has been shown in women treated with clindamycin for BV and AV (Lamont et al., 2003; Larsson et al., 2006). However, multiple large, randomized trials did not find reduced incidence of preterm birth after clindamycin treatment (Bellad et al., 2018; Subtil et al., 2018), but preterm birth rates were much higher in the participants who failed antibiotic treatment (McGregor et al., 1994; Kekki et al., 2001; Sabol et al., 2006). In these cases, antibiotic resistance is likely to occur, and the normal vaginal flora is not maintained. Therefore, the current consensus in the field is that there is no significant reduction in the risk for preterm birth with clindamycin therapy (McClure and Goldenberg, 2019; Smaill and Vazquez, 2019). However, the mixed results may still warrant further investigation. Disruption of the vaginal microbiota has been proven to affect the incidence of preterm birth (Jacobsson et al., 2002). More recent studies of the vaginal microbiome of pregnant women with whole genome shotgun sequencing also reveal the similarities of *G. vaginalis* to the *Bifidobacterium* genera. In contrast, an *L. crispatus*-dominated VMB was significantly correlated to full-term birth, along with *L. gasseri* and *Bifidobacterium breve* (Feehily et al., 2020). However, current evidence shows that the overgrowth of BV-associated bacteria such as *G. vaginalis* generates proteolytic enzymes such as sialic acidase and proline aminopeptidase to break down the protective components of the vaginal tract such as mucin and the vaginal secretions and the fetal membranes (Cauci et al., 2008). The loss of protection allows pathogenic adhesion to the vaginal mucosa and invasion of the uterus, whereas the affected elasticity of the fetal membranes potentially leads to early rupture (Gao et al., 2021). Further, the protective low pH in the vaginal microenvironment is disrupted due to the lack of lactobacilli metabolic activity and concurrent biogenic amine production by BV-associated bacteria, possibly allowing more favorable conditions for opportunistic bacteria and pathogens to grow (Nelson et al., 2015; Borgogna et al., 2021). The parasitic *T. vaginalis* infection occurs in these conditions in symbiotic interactions with *Mycoplasma hominis*, leading to severe complications such as preterm delivery and low birth weight (Dessì et al., 2019; Margarita et al., 2020).

Continued vaginal dysbiosis can also affect the local immune response, leading to preterm birth. Within the mucosal layer, secretory immunoglobulin A (sIgA) antibodies and immune cells such as macrophages, B and T lymphocytes are present (Gomez-Lopez et al., 2017). However, with the process of infection during upstream movement or within the disrupted amniotic cavity, bacterial endo- and exotoxins generated further aggravate the condition by the stimulation of increased levels of proinflammatory cytokines such as IL-1α, IL-1β and TNF-α (Bogavac et al., 2010; Gao et al., 2021). Such conditions could potentially dysregulate hormonal balance which trigger the body’s synthesis of prostaglandin and matrix metalloenzymes leading to early contractions and dilation of the cervix, and destruction of collagen matrices that maintain the fetal membranes (Bogavac et al., 2010; Triggs et al., 2020; Gao et al., 2021). Incidentally, one bacterium gaining notoriety in association with adverse pregnancy outcomes involves the opportunistic pathogen *Fusobacterium nucleatum*, as it has been detected in placental and fetal tissues in pregnancy complications involving both ruptured and intact fetal membranes (Han et al., 2010; Bohrer et al., 2012; Wang et al., 2013). Even though it is initially an oral and vaginal commensal, it has been shown to have invasive properties in murine models that bind and infect epithelial and endothelial cells of the fetal-placental units, with TLR4 inflammatory cascades that lead to poor outcomes such as preterm birth, chorioamnionitis, neonatal sepsis, stillbirth, and preeclampsia (Han et al., 2004; Vander Haar et al., 2018). Studies in mice also show potential glycan cross-feeding mechanisms between *F. nucleatum* and BV-associated bacteria, potentiating sialidase activities in the dysbiotic vaginal microbiome (Agarwal et al., 2020).

Likewise, BV-associated bacteria have been linked to increased susceptibility in acquiring more severe inflammatory infections from sexually transmitted infections affecting the upper female genital tract, including the cervix, uterus, fallopian tubes, and ovaries (Wiesenfeld et al., 2002; Edwards et al., 2019). BV-induced pelvic inflammatory disease is a risk factor for ectopic pregnancies, and chronic infections can severely affect the reproductive organs, resulting in infertility and low success rates with IVF treatment (Haahr et al., 2016). Thus, the endometrial microbiome became another center of focus. Although lower in biomass as compared to the vaginal microbiome, the commensal endometrial microbiome possibly supports the intricate balance that regulates embryo implantation, a natural inflammatory event, and pregnancy development (Odendaal et al., 2024). Despite the technical and sampling limitations, it is necessary to further understand the influence of the cervicovaginal microbiota and utilize restorative LBP therapies such as lactobacilli, to resolve such inflammatory infectious or even non-infectious gynecological conditions, and to improve maternal health by treating vaginal dysbiosis in reproductive-aged women (Haahr et al., 2022).

## LBP mechanisms of action and screening for desired characteristics

**Figure 2:**
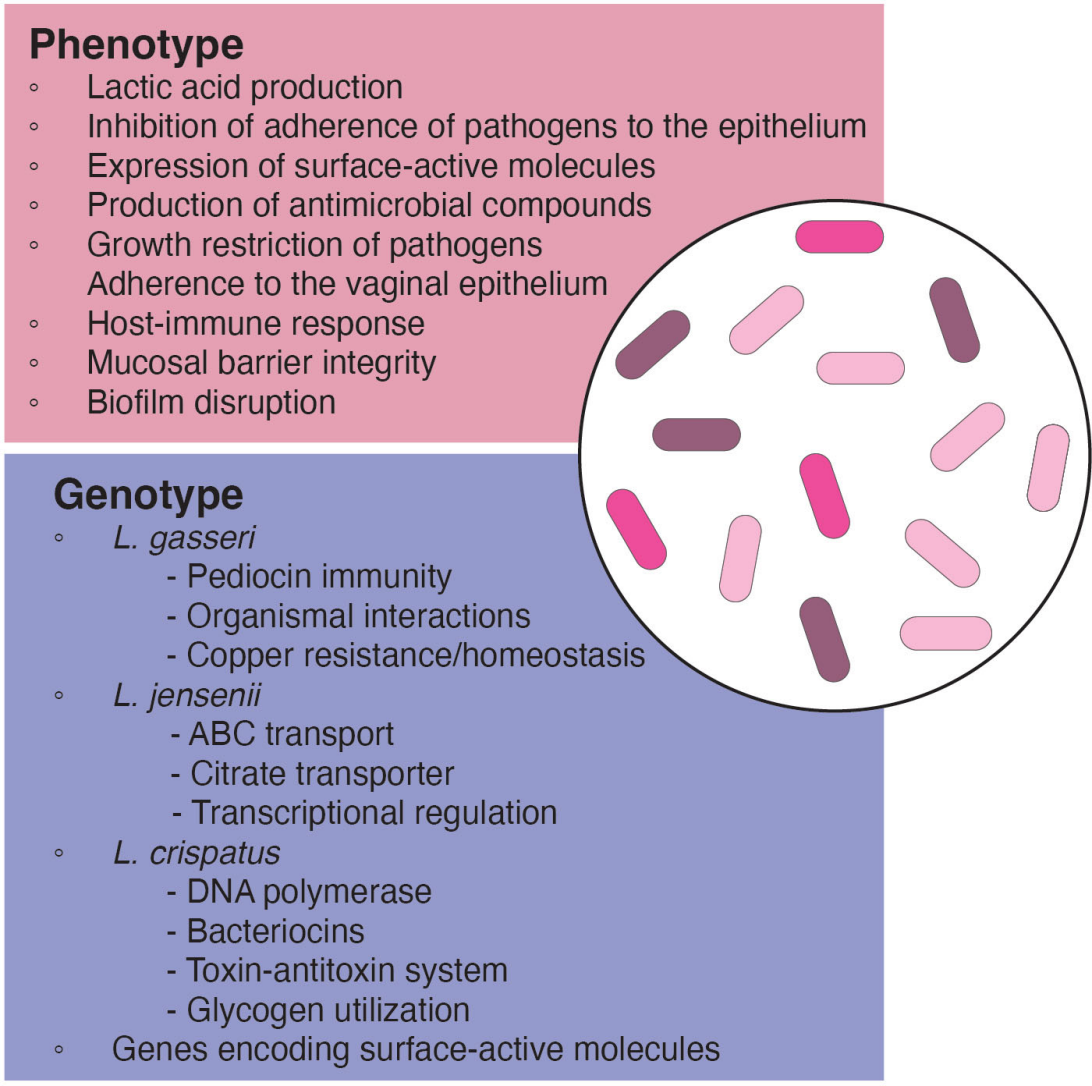
Phenotypic and genotypic characteristics of relevant species and strains for use in LBP development.

Beneficial and antipathogenic effects of lactobacilli for LBP use are based on the traditional probiotic traits of lactobacilli such as in the production of lactic acid to maintain a low vaginal pH, production of antimicrobial compounds (i.e., bacteriocins, hydrogen peroxide) and stimulation of the immune system to help to maintain bacterial balance in the vaginal tract (López-Moreno and Aguilera, 2021). In addition, by adhering to the vaginal epithelia, vaginal lactobacilli may inhibit the attachment of pathogenic bacteria and utilize the same nutrients as pathogens, thereby restricting their growth (Lehtoranta et al., 2022). Given so, an understanding of the local vaginal *Lactobacillus* species and their niche provides insight into the selective criteria for LBP development (**Figure 2**).

### Ecological fitness, competence and growth

Lactobacilli display distinct strain differentiation in both genotypic and phenotypic characteristics within the same species. Within vaginal species, each genome encodes species-specific protein families. Among the most common vaginal lactobacilli—*L. crispatus*, *L. gasseri*, *L. iners*, and *L. jensenii*—*L. iners* exhibited the smallest average genome size at 1.3 Mbp, lacking integral membrane proteins, transcriptional regulators, acetyltransferase GNAT family members, and certain ABC transport proteins. However, they possessed unique ABC transporter permeases, thiol-activated cytolysin (inerolysin), and other protein families absent in other *Lactobacillus* species (Rampersaud et al., 2011; Macklaim et al., 2013). On the other hand, *L. crispatus*, known for its extensive genome size among vaginal lactobacilli, exhibited unique proteins such as DNA polymerase, bacteriocins, and toxin-antitoxin systems, absent in other vaginal strains. Generally, *L. crispatus* harbored all common protein families with the other *Lactobacillus* species, but no protein families were significantly more abundant in *L. crispatus* (Damelin et al., 2011). For *L. gasseri*, proteins related to copper resistance/homeostasis, toxin-antitoxin systems, and pediocin immunity were exclusive across all tested strains. In the case of *L. jensenii*, protein families involving families associated with ABC transport, transcriptional regulation, and a citrate transporter were unique. However, certain protein families such as the glucitol/sorbitol phosphotransferase system were present in other vaginal lactobacilli but were missing in *L. jensenii* (Merhej et al., 2009; Mendes-Soares et al., 2014).

Pan et al. (2020) also compared phenotypic assays of *L. crispatus* and *L. gasseri* in healthy vaginal microbiomes and human gastrointestinal tracts. Variations in the growth patterns and fermentative capacities are observed within the same species, where vaginal lactobacilli performed well in a simulated vaginal fluid, demonstrating higher lactic acid resistance at a later growth phase was necessary for the maintenance of low vaginal pH. Their findings underscored that the isolation source of a strain could influence its potential probiotic functionality, emphasizing its consideration in probiotic formulation (Pan et al., 2020). Similarly, clinical evidence shows that specific oral probiotic strains or their combinations may elevate vaginal lactobacilli counts in healthy women or women with BV and/or VVC and support natural VMB during/after recovery from antibiotics/antifungal treatment (De Alberti et al., 2015; Heczko et al., 2015; Mezzasalma et al., 2017). Nevertheless, current clinical research also shows that this has not always been the case in other BV, GBS infection and VVC cohorts, where there was no consistent improvement and vaginal colonization of orally administered probiotic strains (Hanson et al., 2014; Husain et al., 2020; Farr et al., 2020). As far, it seems that given the longer length, higher dosing, and necessity for lactobacilli to withstand gastric and bile acids for treatment via the oral route, the direct intravaginal route of antibiotic and LBP therapy is deemed more effective, relevant to the source of the strain.

Analysis of *L. crispatus* strains from various environments revealed genetic adaptations of strains to their ecological niches. Certain well-adapted metabolic and growth capacities to the vaginal tract can provide an advantageous basis for colonization and provide means for restoration of the natural vaginal lactobacilli. Notably, a vaginal isolate, *L. crispatus* PRL2021, demonstrates superior ecological competence in a simulated vaginal fluid compared to other vaginal *Lactobacillus* species, indicating its potential in higher growth performance in the vaginal microbiome and competitive mechanisms (Mancabelli et al., 2021). *L. crispatus* strains from *Lactobacillus*-dominated and dysbiotic vaginal microbiomes were also studied, but with no phenotypic distinction observed between strains, specifically with organic acid production (Abdelmaksoud et al., 2016; van der Veer et al., 2019). However, they observed a disproportionally higher abundance of gene fragments encoding for glycosyltransferases among strains isolated from dysbiotic microbiomes, suggesting a role for cell surface glycoconjugates in shaping vaginal microbiota-host interactions. Additionally, they noted the ability of *L. crispatus* to grow on glycogen, correlating with the presence of a full-length pullulanase type I gene (van der Veer et al., 2019). In all studies, glycogen utilization deemed favorable for selection as it is the most abundant carbohydrate source in the vaginal milieu, supporting both growth and lactic acid production.

### Antimicrobial production

Lactic acid has both antimicrobial and immune modulatory functions. It does not only acidify the vaginal environment but is also associated with lower levels of pro-inflammatory cytokines (e.g., IL-1α, IL-6, IL-8, and TNFα), which help to generate a homeostatic environment (Aldunate et al., 2015). Several *in vitro* studies have demonstrated that lactic acid is a potential antiviral and bactericidal compound, inhibiting replication/growth of genital STI pathogens and pathobionts, including *C. trachomatis*, *N. gonorrhoeae*, GBS, HPV and HIV (Muzny et al., 2020). In this context, acidity and protonated lactic acid are responsible for the anti-trichomonadal effects of *L. gasseri* (Pradines et al., 2022) alongside other lactobacillar bacteriostatic and bactericidal compounds in the vagina (Brotman et al., 2012). Other antimicrobial compounds such as hydrogen peroxide-producing enzymes are common among vaginal *L. crispatus* strains (van der Veer et al., 2019). The rationality of hydrogen peroxide production as a significant factor in vaginal health has been debated due to the lack of oxygen in the healthy vaginal tract (Tachedjian et al., 2018), and that physiological concentrations of hydrogen peroxide (<100 uM) do not affect BV bacteria (O’Hanlon et al., 2011). Nevertheless, given certain physiological factors causing vaginal dysbiosis such as in AV and VVC, it may still be relevant as the natural vaginal strains still maintain the core functional genes. In this context, vaginal *L. crispatus* supernatants are strongly inhibiting of *Candida albicans* growth, virulence gene expression and hyphal formation (Wang et al., 2017). Furthermore, bacteriocins have also been found in certain vaginal lactobacilli. Lactocin 160 from a vaginal *L. rhamnosus*, induces transient pore formation via disruption of the chemiosmotic potential on the cytoplasmic membrane of *G. vaginalis* (Turovskiy et al., 2009), whereas Gassericin E from *L. gasseri* EV1461 affects both related and non-related strains, inhibiting the growth of BV pathogens (Maldonado-Barragán et al., 2016).

### Modulation of host innate and adaptive immune responses

Further, LBP host colonization and the inhibition of pathogen adhesion are important selective traits for maintaining mucosal barrier integrity (Boris et al., 1998; Osset et al., 2001; Delgado-Diaz et al., 2022). Lactobacilli can induce host defense against pathogens through the formation of microcolonies that attach to epithelial cell receptors and form a physical barrier to pathogen attachment (Lebeer et al., 2010; Petrova et al., 2013). For instance*, L. gasseri* inhibits *T. vaginalis* colonization of vaginal epithelial cells through the action of its cells and a cell-surface aggregation-promoting factor (Pradines et al., 2022). Similarly, genomic studies of *L. crispatus* also found genes encoding fibronectin-binding *L. crispatus* protein (LACT01268) and *L. crispatus* adhesins (LACT01712 and LACT02327) that have been found to interfere with fibronectin-binding and pilus components of *G. vaginalis* (Koumans et al., 2007; Yeoman et al., 2010). One such fibronectin-binding protein encoded by *L. crispatus* the LEA protein, which can potentially exert inhibitory effects by competing with the same attachment sites as the pili of *G. vaginalis* (Edelman et al., 2012; Ojala et al., 2014). Core proteins of *L. crispatus* may play a crucial role in protecting the vagina from pathogens and bacterial vaginosis and highlight intricate mechanisms through which *L. crispatus* maintains vaginal health.

At the same time, potential LBPs demonstrate the ability to improve host innate immune responses. TLRs on vaginal epithelial cells regulate the production of cytokines by responding to molecular patterns (MAMPs) present on the bacterial surface. For instance, *Gardnerella* can induce an epithelial cell response through nuclear factor kappa B (NF-κB) activation via a TLR2-dependent signaling pathway (Anton et al., 2022). In AV, inflammatory responses can also be induced through TLR4 activation of NF-κB (Mirmonsef et al., 2011; Rose et al., 2012). Given this, TLR2/TLR6 modulation has been suggested to play an anti-inflammatory role in certain situations (Li et al., 2013). To ameliorate the low activation of TLR2/TLR6 typically observed in an inflammatory state, *Lactobacillus*-dominated commensal communities may be an important part of maintaining homeostasis and inducing an immunomodulatory role to reduce vaginal inflammation (Nasu and Narahara, 2010; Hearps et al., 2017). Surface active molecules such as bacterial exopolysaccharides (EPS) and peptidoglycan on the cell surfaces are also present in *L. crispatus* (Ojala et al., 2014; Chee et al., 2020). EPS can enhance the ability of vaginal VK2 cells in producing anti-candidal human defensin-2 protein, and likewise improving colonization potential (Donnarumma et al., 2014). *In vitro* evidence also shows the ability of the vaginal *L. crispatus* peptidoglycan to stimulate CD207 expression in Langerhans cells, the antigen presenting dendritic cells in vagina, to reduce HIV receptor entry (Song et al., 2018).

Lastly, it has been demonstrated that certain *Lactobacillus* species such as *L. delbrueckii* and *L. rhamnosus* taken as oral probiotics may dampen lipopolysaccharide (LPS)-induced expression of HLA-DR, CD86, CD80, CD83, and IL-12 in human dendritic cells (Esmaeili et al., 2018; Odendaal et al., 2024). Although it is not yet well understood, it may be a process through which the microbiota modulates immunotolerance in early pregnancy (Inversetti et al., 2023; Odendaal et al., 2024). Nevertheless, further investigation is needed to explore the mechanisms associated with local immunomodulation within the cervicovaginal microbiome, and their relevance to the implantation regulation and pregnancy development.

## Currently available LBPs and ongoing developments

### Traditional probiotics vs. LBPs

In the US, alternative treatment options are classified only as vaginal probiotics, due to the more straightforward track for getting them out to the market. Currently, there are seven different vaginal probiotics available for BV support. The AZO Complete Feminine Balance™ and Jarro-Dophilus^®^ Women contain similar strains of *L. crispatus* LbV 88, *L. jensenii* LbV 116, *L. gasseri* LbV 150N, and *L. rhamnosus* LbV 96, which are a mix of the most common *Lactobacillus* spp. found in the vagina and has been documented to have increased recovery rates from BV (Laue et al., 2018). On the other hand, Bio-K+^®^ Women’s Health, Jarrow Formulas^®^ Fem-Dophilus^®^ *1 Billion*, Jarrow Formulas^®^ Fem-Dophilus^®^ *5 Billion*, RepHresh™ Pro-B™ Probiotic, and UltraFlora^®^ Women’s utilize strains isolated from the urogenital tract, such as *L. reuteri* RC-14 and *L. rhamnosus* GR-1, which did not improve BV cure rates as a dietary supplement, but can contribute to improved vaginal flora composition (Hummelen et al., 2010).

Moving forward, it is crucial to consistently differentiate between using *Lactobacillus* strains as a traditional probiotic and its use as an LBP. More relevant regulatory functions and expectations are necessary for the drug development track, considering that the target populations are diseased individuals who may be immune-sensitive (Cordaillat-Simmons et al., 2020). As far, *Lactobacillus* bacteremia is rare and mainly correlated to intestinal translocation in severely immunocompromised patients (i.e. uncontrolled diabetes mellitus, malignancy or have undergone organ transplantation), urogenital pathologies, congenital and valvular heart diseases or those who have undergone invasive procedures, that may be more susceptible to probiotic use (Salminen et al., 2004; Rossi et al., 2022; Salminen et al., 2006; Bapna et al., 2023; Kullar et al., 2023). There are currently no clinical reports of *Lactobacillus* bacteremia with intravaginally administered LBP strains, but vaginal lactobacilli, specifically *L. jensenii*, has been reported in polymicrobial bacteremia with *Veillonella montpellierensis* during pregnancy in an immunocompetent but anemic patient at 33-weeks of gestation (Toprak et al., 2022). Another recent case involves *L. jensenii* and *P. bivia* polymicrobial bacteremia leading to renal and perinephric abscesses with an otherwise immunocompetent patient but has undergone ureteral stent procedure (Mohan et al., 2020). Other case reports of *L. jensenii* bacteremia within obstetrics and gynecology that have occurred after vaginal delivery (Marciniak et al., 2014) and elective abortion using dilatation and curettage (Suárez-García et al., 2012) with patients who presented with infective endocarditis.

Nevertheless, the use of lactobacilli as a biotherapeutic drug is still justifiable due to its lower risk of use as a “generally regarded as safe” (GRAS) microorganism and its positive benefit-risk ratio compared to antibiotic usage in patients suffering from recurrent vaginal infections. Likewise, such side effects and contraindications can be averted with a well-characterized LBP strain lacking pathogenic factors and a defined antimicrobial sensitivity spectrum. These LBP candidates should be further explored within the ongoing clinical trials. Nevertheless, same as other drugs, high doses should also be used with caution, and monitored carefully by the administering physician along with any presenting underlying illnesses.

Thus, vaginal lactobacilli developed as intravaginally administered LBPs are meant to undergo further trait selection and safety testing, including considerations of the proper dosing, delivery form and optimal administration frequency and route, therapeutic indications, and expected side effects. Further, strain-specific properties and treatments prior to LBP administration is crucial (Wu et al., 2022). Based on this, various treatment modalities have been considered, such as the use of single lactobacilli strains, multi-strain lactobacilli combinations, and vaginal microbiome transplantation (VMT), with varying levels of efficacy specifically against BV, with or without antibiotic therapy prior to LBP treatment.

### Single strain LBPs vs. multi-strain LBPs in recurrent BV and VVC

Substantial research has explored LBPs for treating recurrent BV and VVC, but they are not yet a standard clinical approach due to differences in trial size, methods, and uses. Recurrent BV has been the most impactful in the health and economic sectors, especially with its associations with diseases such as HPV, HIV and *Trichomonas* infection. Thus, clinical trials utilizing strains native to vaginal microbiome are on their way for recurrent infections. Those with clinical trial IDs, are intravaginally administered, and have published results are summarized in **Table 1**.

**Table 1:** List of published clinical trials of intravaginally-administered adjuvant probiotic therapy and LBP development.

Title	Year	Clinical Trial ID	Study type	Enrollment (Actual)	Time Frame	Composition	Dose	Inclusion criteria	Condition	Phase	Aim	Result	References
Exploratory Study to Evaluate the Effects of the Probiotics *L. rhamnosus* GR-1 and *L. reuteri* RC- 14	2012- 2013	NCT02139 839	DB, R, C, PC	14	40 days	*L. rhamnosus* GR-1/*L. reuteri* RC-14	2.5×10^9^ CFU/strain/ capsule	Age: 40-80 years, Other: in good general health, but excluding participants with a Nugent Score of 0 3 or greater than 6	BV, Menopause	Early Phase 1	The objective of this double blinded, placebo-controlled crossover study was to evaluate in 14 post- menopausal women with an intermediate Nugent score, the effect of 3 days of vaginal administration of probiotic *L. rhamnosus* GR-1 and *L. reuteri* RC-14 on the microbiota and host response.	The probiotic treatment increased abundance of *Lactobacillus* and lactate levels in the vagina, along with decreased abundance of *Atopobium*. Microarray results showed probiotics modulate immune responses via TLR2, affecting epithelial barrier function.	Bisanz et al., 2014
A prospective, placebo- controlled, double-blind study for the investigation of the effect and safety of EcoVag® as adjuvant treatment after treatment with clindamycin against bacterial vaginosis	2004- 2006	ISRCTN628 79834	DB, R, PC	100	6 months	*L. gasseri* (Lba EB01- DSM 14869), *L. rhamnosus* (Lbp PB01- DSM 14870)	10^8^-10^9^ CFU/strain/ capsule	Age: 18-60 years, Other: Amsel criteria for bacterial vaginosis	BV	NA	The primary objective of this study was to investigate if supplementary lactobacilli treatment could improve the initial cure rate after vaginal clindamycin therapy, and secondly, if lactobacilli as repeated adjunct treatment during 3 menstrual cycles could lengthen the time to relapse after initial cure.	In the lactobacilli group, the initial intent-to-treat analysis showed a one- month cure rate of 64%, comparable to the placebo group at 78% However, considering population changes and excluding non-compliant patients, the adjusted initial cure rate for the lactobacilli group was 77%. Over six menstrual cycles, lactobacilli-treated women exhibited a significantly higher BV-free rate (64.9%) compared to the placebo group (46.2%), with a significant difference in the time from cure to relapse favoring lactobacilli treatment. Adjuvant therapy with lactobacilli significantly contributed to relapse avoidance.	Larsson et al., 2008
Will *Lactobacillus* Increase Cure Rate After Treatment of Bacterial Vaginosis and Chronic Vulvovaginal *Candida*	2014- 2015	NCT02295 579	0	50	6 months	*L. gasseri* (DSM 14869) *L. rhamnosus* (DSM 14870)	10^8^ CFU/strain/ capsule	Age: 18-55 years, Other: Diagnosis of bacterial vaginosis according to Hay/Ison or with candidiasis diagnosed with wet smear.	BV, R-VVC	NA	The aim of this study was to investigate the colonisation by lactobacilli and clinical outcome in women with BV and recurrent-VVC (R-VVC) receiving antibiotic or anti- fungal treatment in combination with the probiotic EcoVag®capsules.	In trial I, BV was treated with clindamycin/metronidazol e followed by 5-day EcoVag® capsules. In trial II, three groups were included: BV with prolonged clindamycin/metronidazol e and EcoVag®, R-VVC with extended fluconazole and EcoVag®, and fluconazole- only treatment. In trial I, the 6-month cure rate for BV was 50%, and in trial II, both the 6- and 12-month cure rates were 67%. For VVC, women receiving fluconazole and EcoVag® had 100% and 89% cure rates at 6 and 12 months, while those with fluconazole only had rates of 100% and 70% *Lactobacillus* species were associated with BV cure in both trials, while EcoVag® strains were significant only in trial II. A change in sexual partner correlated with BV relapse.	Pendharkar et al., 2015
Study of the Vaginal Microbiota and the Potential of a Vaginal Probiotic Cream in Vaginal Candidosis	2016- 2019	NCT03975 569	INT, FT	20	4 weeks	*L. plantarum* WCFS1, *L. pentosus* KCA1 and *L. rhamnosus* GG	10^9^-10^10^ CFU/g	Age: 18 50 years, Other: positive *Candida* microscopy and/or culture, at least two of the following vaginal symptoms: burning, itching, redness, fissure, discharge, vulvar edema, postcoital itching, lesions with partner	VVC	NA	Here, three strains were selected for further evaluation in patients based on *in vitro* anti-*Candida* effects. They showed their ability to inhibit *Candida albicans in vitro* and formulated them in a gel, **which was tested in a proof- of-concept study aimed at investigating their influence on the vaginal microbiome and highlighting relevant characteristics for future improvements for probiotic strategies against WC.	45% of women using the probiotic gel experienced symptom relief without needing additional fluconazole medication, and their fungal concentrations were comparable to those treated with fluconazole. Fluconazole alone reduced the abundance of vaginal lactobacilli.	Oerlemans et al., 2020b
Probiotic Implementation as Help in Solving Vaginal Infections	2015- 2016	NCT03372 395	R	117	9 months	*L. rhamnosus* BMX 54	10^4^ CFU/tablet	Age: 18 years and older, Other: documented BV or yeast vaginitis associated with HPV-infection documented as PAP-smear abnormalities (ASCUS, L-SIL or H-SIL histologically demonstrated as CIN1) and/or positive for HPV-DNA	BV	Phase 2	The aim of this study was to confirm that vaginal lactobacilli long-lasting implementation in women with HPV-infections and concomitant bacterial vaginosis or vaginitis might be able to help in solving the viral infection, by re- establishing the original eubiosis.	Individuals in the long-term probiotic usage group (Group 2) had twice the likelihood of resolving HPV-related cytological anomalies compared to those in the short probiotic usage group (Group 1). A total clearance of HPV was observed in 11.6% of short-term probiotic users compared to 31.2% in long-term users based on a negative HPV-DNA test at the end of the study period.	Palma et al., 2018
Clinical and microbiological efficacy of new probiotics in vaginal discharge disturbances	2016- 2019	ISRCTN348 40624	R, P	182	3 months	*L. crispatus* strains DSM32717, DSM32720, DSM32718 and DSM32716	10^10^ CFU/strain/ capsule	Age: 18 50 years, Other: Recurrent-BV (R-BV) episodes (diagnosed with complaints and through Amsel criteria, confirmation with microscopy in 2 days) or recurrent VVC (R-VC) episodes (diagnosed with complaints and clinical signs confirmation with culture testing in 2 days)	R-BV, R- VVC	NA	The aim of the current study was to evaluate the activity of the evidence-based probiotics (*L. crispatus* strains DSM32717, DSM32720, DSM32718 and DSM32716) on BV and VVC patients in the clinicaltrial in terms of both clinical and microbiological improvement.	Both oral and vaginal capsules effectively improved symptoms in patients with BV, including reducing discharge volume, odor, and itching/irritation. In VVC patients, both types of capsules successfully alleviated the main symptoms of discharge volume and itching/irritation.	Mändar et al., 2023
Intravaginal LACTIN-V for Prevention of Recurrent Urinary Tract Infection	2006- 2012	NCT00305 227	DB, R, PC	100	10 weeks	*L. crispatus* CTV-05	10^8^ CFU/mL	Age: 18 50 years, Other: current symptomatic uncomplicated cystitis, and the cystitis is treated with trimethoprim- sulfamethoxaz ole (TMP-SMX)	UTI	Phase 2	Major goals of the study were to assess the ability of the probiotic to reduce the incidence of rUTI, to evaluate whether the probiotic achieved vaginal colonization, to assess effects on the vaginal microbiota of women after treatment for UTI, and to confirm the safety of the probiotic.	Women receiving Lactin-V experienced a lower recurrence of UTIs compared to those on placebo. Colonization with *L. crispatus* significantly reduced recurrent UTIs only in those treated with Lactin-V.	Stapleton et al., 2011
Safety and Efficacy Study of *Lactobacillus* Administered Vaginally in Women with Bacterial Vaginosis	2008- 2009	NCT00635 622	DB, R, PC	24	28 days	*L. crispatus* CTV-05	2x10^9^ CFU/dose	Age: 18-40 years, Other: Untreated BV (asymptomatic or symptomatic) as diagnosed during the screening visit using Amsel criteria AND confirmed in the laboratory using the Nugent scoring system (Nugent Score 7)	BV	Phase 2a	This phase 2a trial was designed to determine the colonization efficiency, safety, tolerability, and acceptability of LACTIN-V of 2 x 10° CFU/dose administered by vaginal applicator in women treated for BV.	In the LACTIN-V group, 61% of women were colonized with *L. crispatus* CTV-05 at Day 10 or Day 28, with 78% colonization among those with complete adherence. Adverse events (AEs) were mostly mild or moderate (90%), evenly distributed between LACTIN-V and placebo, and predominantly of genitourinary origin (78%). No severe AEs occurred, and the product was well- tolerated without deep epithelial disruption during colposcopic evaluation.	Hemmerling et al., 2010
LACTIN-V Study for Recurrent Bacterial Vaginosis	2016- 2019	NCT02766 023	DB, R, PC	228	10 weeks	*L. crispatus* CTV-05	2x10^9^ CFU/dose	Age: 18-45 years, Other: Untreated BV (asymptomatic or symptomatic) as diagnosed during the screening visit defined by >/=3 Amsel criteria, and as confirmed in the laboratory using the Nugent scoring system (Nugent Score >/= 4)	BV	Phase 2b	A randomized, double-blind, placebo-controlled, phase 2b trial to evaluate the ability of *L. crispatus* CTV-05 (Lactin-V) to prevent the recurrence of bacterial vaginosis.	The recurrence of BV by week 12 was lower in the Lactin-V group compared to the placebo group. *L. crispatus* CTV-05 was detected in 79% of Lactin- V participants at the 12- week visit, and adverse events were similar between Lactin-V and placebo groups.	Cohen et al., 2020

DB (Double Blind), RCT (R), C (Crossover), P (Parallel), PC (Placebo Controlled), O (Observational), INT (Interventional), FT (Fluconazole Treatment); BV (Bacterial Vaginosis), VVC (Vulvovaginal Candidiasis), UTI (Urinary Tract Infection); NA (Not applicable).

Early phase 1 trials have shown that vaginal administration of *L. rhamnosus* GR1 and *L. reuteri* RC-14 can increase vaginal *Lactobacillus* and modulate the immune response via TLR2 (Bisanz et al., 2014). EcoVag^®^ with *L. gasseri* DSM 14869 and *L. rhamnosus* DSM 14870 showed possibility for adjunct therapy during three menstrual cycles, demonstrating a higher BV-free rate (64.90%) in the lactobacilli group compared to placebo group (46.2%) (Larsson et al., 2008). EcoVag^®^ also shows strong potential in recurrent VVC alongside fluconazole treatment, but with only a 6- or 12-month cure rate of 50 and 67%, respectively, alongside clindamycin/metronidazole treatment for BV, respectively (Pendharkar et al., 2015). *L. plantarum* WCFS1, *L. pentosus* KCA1 and *L. rhamnosus* GG administered as a probiotic gel against VVC also achieved 45% symptom relief in their interventional therapy (Oerlemans et al., 2020b). Lastly in a phase 2 trial, *L. rhamnosus* BMX54 showed long-term use benefits for HPV clearance, in the context of vaginal dysbiosis (Palma et al., 2018).

As far, it is still debatable if the effect may be dependent on *Lactobacillus* species or their natural ecological niche, as similar reference strain standards have been utilized. However, given that most studies are interventional with BV recurrence, larger clinical cohorts are still lacking. Further, long-term effects are only found in a few of these studies, and few have been done without the use of antibiotics; thus, the effect on varying vaginal microbiomes, retention, and mechanisms of action of these probiotic strains developed as potential LBP candidates may still need elucidation as the LBP drug functionality is still lacking in most studies.

Nevertheless, it has generally been accepted that native strains are more competitive and well-adapted to their ecological niche, such as in the use of glycogen in the vaginal microenvironment as a carbon source. Most lactobacilli are not capable of utilizing glycogen directly, but native vaginal strains have a generally efficient acquisition of glycogen-derived resources released by α-amylase production that break down glycogen in the vaginal tract (Nunn and Forney, 2016). Notably, LBPs containing native vaginal microbiome strains also stand out for their potential benefits in biofilm disruption and immunomodulation (Łaniewski and Herbst-Kralovetz, 2021). Exciting new prospectives for using natural, non-antibiotic pre-treatment alternatives, potentially in combination with LBPs, lie ahead (Latham-Cork et al., 2021).

Extensive research highlights *L. crispatus* as advantageous, providing protection against various pathogens and correlating with positive health and pregnancy outcomes (Ghartey et al., 2014; Argentini et al., 2022; Baud et al., 2023). Furthermore, compared to other dominant lactobacilli strains, pangenomic gene analyses have shown greater enrichment and evolutionary acquisition of key beneficial and protective functional genes (Bhattacharya et al., 2023). Moreover, although bacteriocin and organic acid biosynthesis are still strain-specific, *L. crispatus* seem to more ubiquitously carry key functionalities as compared to the heterogeneity found in either *L. iners*, *L. gasseri* or *L. jensenii* (Bhattacharya et al., 2023).

*L. crispatus* strains DSM32717, DSM32720, DSM32718 and DSM32716 which come from a collection of reproductive tract microorganisms, have been phenotyped and observed to be effective in reducing BV and VVC symptoms using both oral and vaginal capsules (Mändar et al., 2023). However, as far, Lactin-V based on the single strain *L. crispatus* CTV-05, has been the only one that has reached clinical phase 3, recently showing relative success in their clinical phase 2 trials for recurrent urinary tract infections in non-pregnant women (Stapleton et al., 2011), and in their phase 2a, and phase 2b trials for recurrent BV applications (Hemmerling et al., 2010; Cohen et al., 2020). In the studies, a 78% colonization rate in women treated for BV, and a lower BV recurrence after 12 weeks with Lactin-V has been demonstrated (Hemmerling et al., 2010; Cohen et al., 2020). Furthermore, Lactin-V has recently shown to be generally safe and well tolerated in pregnant women (Bayar et al., 2023), and is currently being investigated for use in reducing pre-term birth in women at high risk and enhancing *in vitro* fertilization success rates.

#### Vaginal microbiome transplantation

Aside from Lactin-V, exploratory studies using VMT have been performed for intractable and recurrent bacterial vaginosis in five women, with four achieving full long-term remission without adverse outcomes, despite a repeat VMT for three (Lev-Sagie et al., 2019). This provides promising results and opens more research opportunities based on VMT. In line with this, Freya Biosciences has started exciting work in LBPs based on VMT to improve reproductive outcomes. Two proof-of-concept intervention studies with their FB101 product have been completed, with published results in a patient presenting with severe vaginal dysbiosis and a history of recurrent pregnancy loss (Wrønding et al., 2023). In this antibiotic-free VMT approach, confirming donor strain engraftment followed by a successful pregnancy and delivery of the patient’s vaginal microbiome from 90% *G. vaginalis* to 81.2% *L. crispatus* and 9% *L. jensenii* with just one VMT treatment. It remains, however, that post-VMT treatment, the patient received 75mg daily of acetylsalicylic-acid and low-molecular-weight heparin to resolve antiphospholipid syndrome that can have contributed to the successful pregnancy (Wrønding et al., 2023). The study demonstrates how VMT can be further utilized in other gynecological conditions resulting from abnormal vaginal microbiota and improving reproductive health outcomes. Large-scale randomized, placebo-controlled clinical studies are ongoing, but no results have been published.

The future applications of vaginal LBPs in influencing the cervical and endometrial microbiota have become a major focus in maternal and fetal health. Further understanding of the cervicovaginal microbiota can assist in guiding the treatment of vaginal dysbiosis relevant to endometrial function to improve reproductive health outcomes (Odendaal et al., 2024).

## Regulatory pathways and challenges in the development of vaginally administered LBPs

The microbiome field has dramatically matured over the past decade, but the development and regulatory approval of life biopharmaceutical products pose several challenges, given the complexity of these live products. However, advancements like the approval of LBPs for preventing CDI recurrence (Mullard, 2022) marked a pivotal milestone that remains to be expanded to other indications.

In 2012, the Food and Drug Administration (FDA) was the first regulatory agency to create the category of LBP and to publish a guideline for industry stakeholders entitled “Early Clinical Trials with Live Biotherapeutic Products: Chemistry, Manufacturing, and Control Information” and recently updated in 2016 (FDA (Food and Drug Administration), 2016). The European Directorate for the Quality of Medicines and Healthcare (EDQM) accepted this new category of medical products on the European market in 2019 entitled “EDQM (European Pharmacopoeia) 3053E General Monograph on Live Biotherapeutic Products” (EDQM, 2019). However, LBPs currently have no separate status in the European regulatory framework. Therefore, developers in the EU need to rely on relevant regulatory concepts for biological medicinal products, but with guidelines from the European Medicines Agency based on information from the International Council for Harmonisation of Technical Requirements for Pharmaceuticals for Human Use (ICH) regarding “ICH guideline E8 on General Considerations for Clinical Studies” in 2021 (European Medicines Agency, 2021) and the Committee for Medicinal Products for Human Use (CHMP) for “CHMP guideline on Human Cell-Based Medicinal Products” for scientific guidelines in addressing development, manufacturing and quality control (European Medicines Agency, 2008) and a revised guideline on “First-in-Human Clinical Trials with Investigational Medicinal Products” (European Medicines Agency, 2017). All these regulations allow for comprehensive risk analysis and mitigation strategies, specifically with their intended use (Rouanet et al., 2020).

LBPs, such as individual strains of bacteria, defined consortia, or VMTs, involve intricate biological processes that are not fully known. Regulatory agencies must assess the complex mechanisms of action on both the human host and host microbiome, potential side effects, and long-term safety. The proposed mechanisms of action are generally to interfere with the growth of a pathogenic or potentially pathogenic microorganism in the vagina or to stimulate other potentially beneficial cellular processes because of transient persistence and/or long-term colonization with the microorganisms contained in the LBP (Wu et al., 2022; Lagenaur et al., 2021). Investigational objectives vary concerning prevention versus treatment and stand-alone therapy versus adjunct therapy to antimicrobial or other therapy. With this regard, newly developed and emerging *in vitro* and *in vivo* tools are becoming more available to support LBP risk documentation before early clinical trials. However, there are still challenges regarding quality control and manufacturing of LBP, as ensuring consistent quality and manufacturing processes for biopharmaceuticals is complex. The biological nature of these products potentially has unknown inherent risks and can lead to manufacturing variability. Thus, maintaining product integrity during large-scale production is one of the significant obstacles to overcome.

The early clinical phases are designed to determine tolerability, dosage range, and administration regimen, which depend on the formulation and delivery route. As of current, designing an effective dosage form and delivery system for vaginal administration poses challenges. The dosage form should provide a controlled release and adhere to the vaginal mucosa for optimal absorption. Safety and tolerability studies need to consider the unique environment of the vagina regarding potential irritation, allergic reactions, or other adverse effects. The potential for immune responses to LBP can impact both safety and efficacy, so when appropriate, an “optimal effective dose” and a “safe maximal dose” are necessary to determine the points at which the intended effect is achieved with no or acceptable adverse effects. Thus, developers must assess and manage immunogenicity risks through appropriate study designs and monitoring strategies in the target populations.

Intended study populations vary from healthy individuals who may be at risk for specific diseases, such as infertility or pregnancy complications, to individuals severely afflicted with vaginal infections or dysplasia. Given this, designing appropriate clinical trials for live biopharmaceuticals can be challenging due to unique patient populations, limited historical data, and the need for sensitive endpoints. Likewise, target populations may require repeat treatment or longer-term usage of the LBPs, where biobanking of samples from consenting participants in the different trials and clinical phases may also be necessary (Rouanet et al., 2020). Thus, rigorous, and well-designed clinical trials are crucial for demonstrating safety and efficacy, where confounding factors influential to the data should also be considered. In addition, patient knowledge, acceptance, and adherence to vaginally administered treatments may be influenced by cultural, social, and individual factors and norms. Ensuring user-friendly, comfortable, and discreet administration methods is crucial.

Finally, the rapid advancement of biopharmaceutical technologies may outpace the development of regulatory science. Addressing scientific gaps and uncertainties in evaluating these products is an ongoing challenge for regulatory agencies. Furthermore, LBP developers often seek global approval, requiring coordination with multiple regulatory agencies. Achieving global harmonization in regulatory requirements and standards can be difficult due to varying regional expectations. Addressing these challenges requires collaboration between regulators, industry stakeholders, and researchers. Ongoing dialogue, transparency, and a proactive approach to adapting regulations to scientific advancements are essential for the successful development, approval, and access to LBPs.

## Conclusion and future directions

Understanding the mechanisms by which the vaginal microbiota can influence the progression of gynecological and obstetrical disorders will lead to the development of personalized approaches to shape the microbiota composition and function, aiding prevention, and treatment of diseases. High-quality studies, including functional genomics, are needed to define the composition of the vaginal microbiota and its mechanistic involvement to the different conditions. This includes a detailed mapping of the ecology of the vaginal microbiota in health and disease to understand the interactions between microbes, pathogens and potential treatments based on live biopharmaceuticals. Furthermore, the complex interplay between the human host and vaginal microbes requires a deeper understanding for successful development of vaginal microbiome-directed therapies.

Regulatory approval of VMT, isolated bacterial strains, or defined consortia of strains will depend on the documentation and demonstration of quality, safety, and efficacy to allow for an assessment of the benefit-risk ratio for an intended use. More refined clinical trials with larger cohorts, defined uses, standardized trials, and longer-term follow-ups will also be necessary for maturing the field and the pioneer LBPs in development. The registration of LBPs as drugs will require a close dialogue between developers and regulatory agencies as the regulatory requirements are not fully defined yet. With this regard, accurate use of terminologies can significantly improve the process.
